# Influenza Research Database: An integrated bioinformatics resource for influenza virus research

**DOI:** 10.1093/nar/gkw857

**Published:** 2016-09-26

**Authors:** Yun Zhang, Brian D. Aevermann, Tavis K. Anderson, David F. Burke, Gwenaelle Dauphin, Zhiping Gu, Sherry He, Sanjeev Kumar, Christopher N. Larsen, Alexandra J. Lee, Xiaomei Li, Catherine Macken, Colin Mahaffey, Brett E. Pickett, Brian Reardon, Thomas Smith, Lucy Stewart, Christian Suloway, Guangyu Sun, Lei Tong, Amy L. Vincent, Bryan Walters, Sam Zaremba, Hongtao Zhao, Liwei Zhou, Christian Zmasek, Edward B. Klem, Richard H. Scheuermann

**Affiliations:** 1 J. Craig Venter Institute, La Jolla, CA 92037, USA; 2 Virus and Prion Research Unit, National Animal Disease Center, USDA-ARS, Ames, IA 50010, USA; 3 Department of Zoology, University of Cambridge, Cambridge, CB2 3EJ, UK; 4 Animal Health Service, Food and Agriculture Organization of the United Nations, Rome 00153, Italy; 5 Northrop Grumman Health Solutions, Rockville, MD 20850, USA; 6 Vecna Technologies, Greenbelt, MD 20770, USA; 7 Bioinformatics Institute, University of Auckland, Auckland 1010, New Zealand; 8 Department of Pathology, University of California, San Diego, CA 92093, USA; 9 Division of Vaccine Discovery, La Jolla Institute for Allergy and Immunology, La Jolla, CA 92037, USA

## Abstract

The Influenza Research Database (IRD) is a U.S. National Institute of Allergy and Infectious Diseases (NIAID)-sponsored Bioinformatics Resource Center dedicated to providing bioinformatics support for influenza virus research. IRD facilitates the research and development of vaccines, diagnostics and therapeutics against influenza virus by providing a comprehensive collection of influenza-related data integrated from various sources, a growing suite of analysis and visualization tools for data mining and hypothesis generation, personal workbench spaces for data storage and sharing, and active user community support. Here, we describe the recent improvements in IRD including the use of cloud and high performance computing resources, analysis and visualization of user-provided sequence data with associated metadata, predictions of novel variant proteins, annotations of phenotype-associated sequence markers and their predicted phenotypic effects, hemagglutinin (HA) clade classifications, an automated tool for HA subtype numbering conversion, linkouts to disease event data and the addition of host factor and antiviral drug components. All data and tools are freely available without restriction from the IRD website at https://www.fludb.org.

## INTRODUCTION

Influenza virus is a major global public health threat. The World Health Organization (WHO) estimates that approximately 5–10% of adults and 20–30% of children are infected by influenza annually (1). Of those, 3–5 million infected individuals experience severe illness resulting in approximately 250 000–500 000 deaths annually. In order to advance influenza virus research, the National Institute of Allergy and Infectious Diseases (NIAID) at the US National Institutes of Health (NIH) is supporting the freely available, web-based Influenza Research Database (IRD) through the Bioinformatics Resource Centers program (https://www.niaid.nih.gov/labsandresources/resources/dmid/brc/). The objective of the IRD resource is to provide a one-stop shop for influenza virus data and analysis tools to drive new discoveries about influenza virus transmission, virulence, host range and pathogenesis, and to develop novel strategies for diagnosis, prevention and therapeutic intervention.

IRD is comprised of three major components:

- a comprehensive collection of influenza virus related data integrated from public archives, data submitters and IRD in-house curation and annotation pipelines, with data types covering sequences and sequence annotations from GenBank (http://www.ncbi.nlm.nih.gov/genbank/) and UniProt (http://www.uniprot.org), immune epitopes from the Immune Epitope Database (IEDB; http://www.iedb.org), 3D protein structures from the Protein Data Bank (PDB; http://www.rcsb.org/pdb), clinical, surveillance and host factor data (2) from direct submissions and curated antiviral drug data from DrugBank (http://www.drugbank.ca);
- a growing suite of analytical and visualization tools customized for influenza virus data analysis, including tools for multiple sequence alignment, phylogenetic tree reconstruction in high performance computing environments, sequence variation determination, metadata-driven Comparative Analysis Tool for Sequences (meta-CATS) (3), BLAST comparison, short peptide identification, PCR primer design, genome sequence annotation, Sequence Feature (4) and Phenotypic Variant Type (PVT) annotation, HA clade classification, HA subtype numbering conversion, surveillance data visualization, protein structure visualization and host factor data enrichment analysis; and
- personal workbench spaces for data storage and sharing.

The growing importance of IRD for influenza research is evidenced by its steadily increasing usage. The number of scholarly articles citing IRD totaled 430 as of August 10, 2016, among which almost half appeared in the last three years and about 20% published in the last 12 months. Furthermore, *Influenza and Other Respiratory Viruses* also reported that the IRD database paper (5) was the #1 cited paper in the journal in 2014. In a survey of research articles that were published in 2011 and had received NIH funding, IRD was the 6th most frequently acknowledged repository for molecular data, behind only some of the major databases managed by the U.S. National Library of Medicine (6). In addition, the IRD website has been heavily used by researchers worldwide, with over 1300 usage sessions per week on average in 2015 as per Google Analytics.

Since its initial launch, the IRD team has continued to improve the resource by adding new features and new data. In this article we highlight the major improvements in IRD since the last publication about IRD in 2012 (5).

## NEW SYSTEM ENVIRONMENTS

### Cloud environment

The IRD user community has been growing continuously. In order to provide faster and more reliable services to the many concurrent users, the IRD infrastructure migrated to the Amazon Web Services (AWS) cloud in July 2016. Through this new cloud environment, we expect to provide higher performance and more stable services to IRD users. Our initial performance test showed that the IRD resource hosted in AWS was ∼2X faster for general database query tasks and ∼6X faster for more complex analyses.

### High performance computing environment

Increased influenza virus surveillance and sequencing efforts worldwide resulted in an increasingly large number of influenza virus sequences and related data, calling for computational infrastructures that can support large-scale data storage and analysis. To meet the needs of computationally intensive analyses, IRD has partnered with the NSF-sponsored Cyber-Infrastructure for Phylogenetic RESearch (CIPRES) Gateway (7) and now provides users with an option to perform computationally-intensive tasks in a high performance computing environment. Specifically, when a user submits a large phylogenetic analysis job to IRD, the user is provided with the option of running the analysis in the CIPRES environment. If the user chooses this option, the sequences needed to generate a tree are sent to CIPRES through an application programming interface (8). Tree calculations are performed in the high performance computing environment, and the resulting tree file is then returned for visualization in the Archaeopteryx tree viewer in IRD (9). An important advantage of the Archaeopteryx implementation in IRD is that it supports user-driven metadata-based tree leaf coloring, which allows for visual pattern recognition in the phylogenetic data. This collaboration between scientific resources allows users to leverage both the high performance computing environment in CIPRES and the metadata-based tree decoration options in IRD.

## NEW FEATURES

### Custom metadata capturing utility

**Figure 1.**
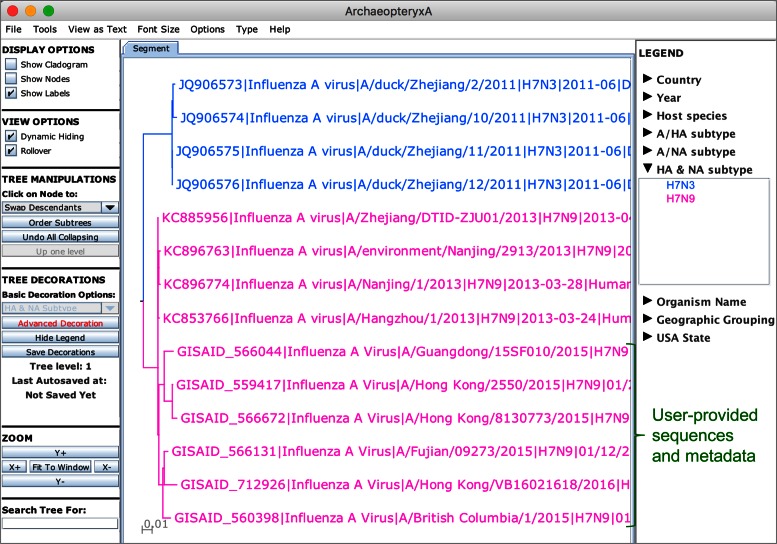
A phylogenetic tree constructed from a combination of user-provided sequences (downloaded from GISAID) and IRD sequences, and visualized in the IRD tree viewer. Tree leaves are color-coded by subtype. The green brace indicates user-provided sequences colored by user-provided HA and NA subtype metadata.

As previously mentioned, one unique capability of IRD is the customized tree viewer, allowing users to color code tree nodes based on sequence-associated metadata, including geographic location, host species, year and season of isolation, HA and NA subtype, H5 clade membership and specific amino acids present at selected protein positions. Previously, this function was only available for trees generated solely from IRD sequences. As this feature became popular, users requested to be able to decorate trees that include their own custom sequences in a similar way. These requests drove the development of a new utility for capturing user-provided metadata. With this new tool, users can provide sequence-associated metadata either in the header of the FASTA sequence file or in a separate metadata spreadsheet. User-provided sequence data and metadata can be further combined with IRD data if desired and then analyzed using any IRD tools. In the case of phylogenetic tree analysis, users can now visualize and decorate a tree based on custom metadata values in the Archaeopteryx tree viewer (Figure 1). Likewise, this new utility allows users to automatically separate sequences by user-provided metadata values for downstream comparison of sequence groups using meta-CATS (3).

### Influenza virus variant protein annotations

**Figure 2.**
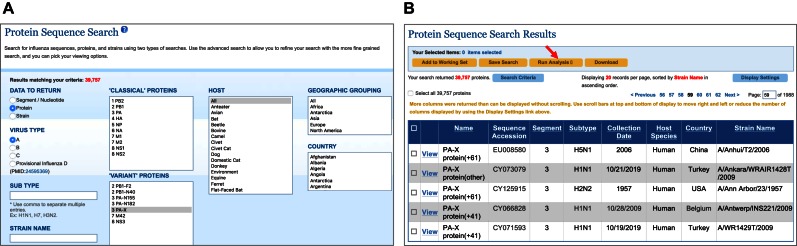
Variant protein annotations in Influenza Research Database (IRD). (**A**) The IRD Protein Sequence Search page supports queries based on ‘classical proteins’, ‘variant proteins’ and sequence-associated metadata. (**B**) A portion of the Protein Sequence Search Results page from a query of PA-X, showing annotations of three PA-X variants: PA-X (+41), PA-X (+61) and PA-X (other). Selected records from this page can be input to any of the analysis tools under the ‘Run Analysis’ dropdown menu (red arrow), or downloaded to a local computer.

In recent years, the influenza community has identified several novel proteins generated from non-canonical translation strategies such as leaky ribosomal scanning (PB1-F2 (10), PB1-N40 (11), PA-N155 (12) and PA-N182 (12)), ribosomal frameshift (PA-X (13)) and alternative splicing (M42 (14) and NS3 (15)). Anticipating the desire to search and analyze these newly discovered variant proteins, the IRD team developed a custom annotation algorithm that predicts the open reading frames and protein sequences for each of the PB1-N40, PA-N155, PA-N182, PA-X, M42 and NS3 variant proteins. Using this algorithm, the IRD team has annotated all relevant influenza segment sequences with variant proteins if they are predicted to be present. These predicted sequences can be retrieved from the Nucleotide Sequence Search and Protein Sequence Search pages (Figure 2A), transferred to any IRD analysis tools (Figure 2B) and downloaded. As of July 2016, over 92% of complete genome strains in IRD have predicted PB1-N40, PA-N155, PA-N182 and PA-X (in three variant forms: +41, +61 or other) proteins (Table 1). M42 and NS3 have very rare and strict alternative splicing, and are therefore only found in 0.2% and 0.1% of influenza strains, respectively. Intriguingly, M42 is predicted to be found in mainly laboratory passaged strains including A/WSN/1933(H1N1) and A/Puerto Rico/8/1934(H1N1), strains from the 1934 highly pathogenic avian influenza Germany outbreak, the 1968 Hong Kong H3N2 outbreak, the 1976 and 1983–1984 North American swine flu outbreaks, the 1986 North American avian H5N2 outbreak, as well as several vaccine strains.

**Table 1.** Variant protein annotations in IRD

Variant Protein	Variant Protein from Complete Genomes	Percentage	Source
PB1-F2	19 701	69.8%	GenBank
PB1-N40	27 909	98.9%	IRD
PA-N155	28 086	99.3%	IRD
PA-N182	26 099	92.2%	IRD
PA-X	27 996	98.9%	IRD
PA-X protein(+41)	8199	29.0%	GenBank & IRD
PA-X protein(+61)	19 721	69.7%	GenBank & IRD
PA-X protein(other)	76	0.3%	GenBank & IRD
PA-X protein	2834	10.0%	GenBank
M42	69	0.2%	IRD
NS3	30	0.1%	IRD

### Phenotype markers and predicted phenotypic effects

In the early stage of IRD development, we developed a novel component for studying genotype-phenotype associations – the Sequence Feature Variant Type (SFVT) component (4). To develop this component, the IRD team compiled a knowledgebase of influenza virus Sequence Features (SFs), where SFs are defined as protein regions with specific interesting structural or functional characteristics. For each SF, all protein sequences in IRD are grouped into individual Variant Types, which are defined by the unique sequence variations existing within the defined SF region. More recently, the SFVT component has been extended to highlight Variant Types that are known to be associated with important phenotypic characteristics.

**Figure 3.**
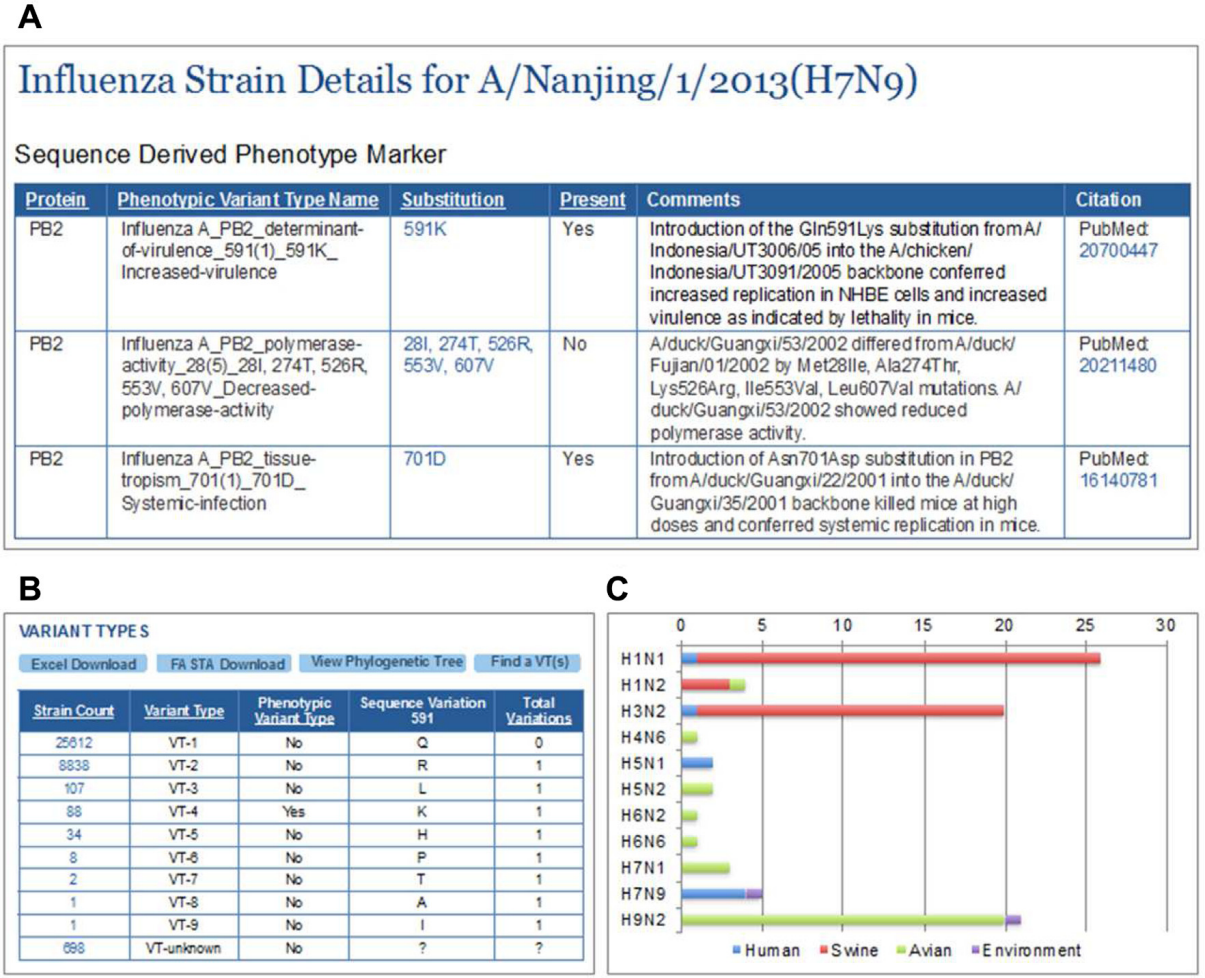
Phenotypic Variant Type (PVT) annotation in IRD. (**A**) A portion of the Strain Details page for A/Nanjing/1/2013 (H7N9) shows that this human isolate carries the PVTs Influenza A_PB2_determinant-of-virulence_591(1)_591K_increased-virulence, which confers increased virulence, and Influenza A_PB2_tissue-tropism_701(1)_701D_Systemic-infection, which confers systemic infection in mouse models, but does not carry Influenza A_PB2_polymerase-activity_28(5)_28I, 274T, 526R, 553V, 607V_Decreased-polymerase-activity, which confers reduced polymerase activity. (**B**) The Sequence Feature Details page for the Influenza A_PB2_determinant-of-virulence_591(1)_591K_increased-virulence showing the SF metadata and variant type (VT) calculation. Within the IRD database, 88 strains, including 8 human strains, carry this PVT (VT-4). The strain count column links to all strains harboring the corresponding VT. (**C**) Host and subtype distribution of VT-4 from panel B.

In response to the highly pathogenic avian influenza H5N1 outbreaks, especially considering the expansion of host and geographic ranges, the WHO Collaborating Center for Influenza Reference and Research and the US Centers for Disease Control and Prevention (CDC) compiled an H5N1 Genetic Changes Inventory (16) to help identify H5N1 strains of concern to cause a potential pandemic. This inventory includes 150 experimentally determined sequence markers associated with a wide range of phenotypic functions: determinant of virulence, tissue tropism, clinical symptoms of disease, replication efficiency, polymerase activity, activation pH, transmissibility, species adaptation, antiviral drug activity, temperature sensitivity, affecting type I IFN pathway and inflammatory response. In order to assist in improving early detection of high-risk H5N1 viruses, we have added these phenotype-associated sequence markers into the SFVT component of IRD. Every relevant protein sequence in IRD has been annotated with these SFs and whether the sequence carries a particular Phenotypic Variant Type (PVT) that is predicted to give rise to a phenotypic consequence (Figure 3A and B). Overall, approximately 66% of influenza strains in IRD contain at least one PVT.

One application of the PVT annotations is to provide guidance for surveillance. In particular, certain PVTs could be used as risk markers for selecting isolates that warrant further investigation. As an example, the PVT of Influenza A_PB2_determinant-of-virulence_591(1)_591K_increased-virulence, which confers increased virulence, is found in 88 strains, mostly from avian strains of H1N1, H3N2 or H9N2 subtypes. However, it has also been found in eight human strains, including subtypes that have caused severe disease in humans such as H7N9 and H5N1 (Figure 3C). Although this PVT is only currently found in less than 1% of strains in IRD, it is important to maintain surveillance of this PVT in the population given its association with disease severity, especially during a pandemic outbreak.

Users can also predict the phenotypic effects of their own sequences by using the SF-PVT annotation tool accessible from the Identify Sequence Features in Segments page.

Moreover, the SF-PVT annotations have been fully integrated with other IRD sequence analysis tools, including the Sequence Variation Analysis and meta-CATS (3) tools. This integration helps users to predict whether a mutation found in an analysis is likely to result in a phenotypic effect.

### Hemagglutinin (HA) clade classifications

IRD development is frequently driven by suggestions from the user community. The United States Department of Agriculture (USDA) Influenza A Virus in Swine Surveillance Program (17) routinely identifies and sequences influenza viruses from the domestic swine population and requested an automated tool that could classify sequences into phylogenetic clades. In collaboration with swine influenza experts at the USDA, the IRD team developed an algorithm to classify the phylogenetic lineages (18) of all North American swine HA (H1) sequences in the IRD database. This algorithm constructs an HA reference tree and then applies the pplacer method (19) to place the query sequence into the reference tree, thus identifying the most closely related lineage of the query sequence. All relevant swine H1 sequences in IRD have now been assigned a clade annotation using this approach. In North America, the most prevalent swine H1 clade is gamma (34.33%), followed by delta1 (24.57%) and beta (13.56%) (Supplementary Table S1). Such clade annotations are searchable via a dedicated Swine H1 Clade Sequence Search page. This classification tool is also available for predicting the H1 clade for user-provided sequences.

A related tool in IRD is an H5N1 clade classification tool. Since 2008, H5N1 viruses in the highly pathogenic avian influenza (HPAI) A/goose/Guangdong/1/1996-lineage have been continuously circulating in isolated geographical regions. To help monitor the evolution of H5N1 virus for its epizootic and pandemic potential, the IRD team developed the H5N1 clade classification tool, which classifies the clade of both highly pathogenic and low pathogenic H5 HA sequences. Similar to the H1 clade classification tool, this algorithm uses phylogenetic analysis to place H5 HA sequences within the WHO classification scheme (20).

All IRD H5 sequences, regardless of host, have been annotated with an H5 clade designation. Most H5 sequences are from Asia, comprising 64% of H5 sequences in IRD and from all documented clades in the H5 phylogenetic tree (Supplementary Table S2). In contrast, H5 sequences from other continents are restricted to certain lineages. For example, in North American, 89% of H5s fall into the American non-Goose Guangdong lineage, while all HPAI H5s (9%) are from the 2014–2015 avian outbreak and classified into clade 2.3.4.4 (highlighted in red). For comparison, most African and European H5s belong to clade 2.2 and its derived lineages, accounting for 91% and 61% of the total H5s, respectively. This suggests that among all HPAI H5s originating in Asia, only certain lineages have migrated to other continents. The H5 annotations can be searched via a tailored H5N1 Clade Sequence Search page. In addition, users can also annotate their own H5 sequences using the H5N1 Clade Classification Tool in IRD.

### HA subtype numbering conversion

There is increased interest in comparing amino acid substitutions across different HA subtypes in order to perform analyses such as comparing amino acids involved in glycan binding by different HA subtypes, comparing substitutions at positions that are associated with other phenotypic and functional changes, and identifying broad range cross-reactive immune epitopes. However, comparing specific residues between different subtypes using sequence-based alignments alone has been challenging. Recently Burke and Smith (21) proposed a cross-subtype HA numbering scheme for the 18 influenza A and influenza B subtypes, using a combination of HA sequence and structural data to propose positions of functional equivalence across the different subtypes. IRD implemented the HA Subtype Numbering Conversion Tool based on this numbering scheme. This tool allows users to convert the coordinates of any HA protein sequence to the corresponding coordinates in any other subtypes. This subtype numbering conversion tool is also integrated with other analysis tools in IRD, including Sequence Variation Analysis and meta-CATS (3), for converting the coordinates of an analysis result into a different coordinate system.

**Figure 4.**
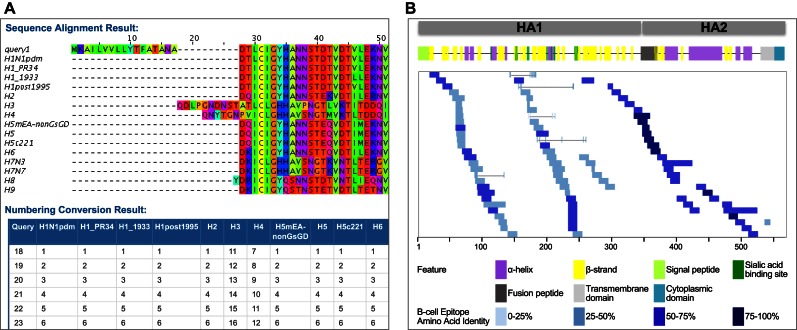
HA subtype numbering conversion in IRD. (**A**) The HA Subtype Numbering Conversion Result page showing the sequence alignment and mapping table for a query H1 sequence mapped into coordinate space for other HA subtypes. The mapping table was used to map all H1 B cell epitopes to all other subtypes. (**B**) A schematic view of all experimentally determined H1 B cell epitopes in the HA protein. Epitopes are colored based on the average percent amino acid identity cross all HA subtypes.

As an example use of this tool, we explored sequence conservation of H1 B-cell epitopes across all HA subtypes. This analysis involved the following four steps: (i) Firstly, we searched for H1 HA B-cell epitopes using the Sequence Feature Variant Type Search page in IRD. In July 2016, this query returned 90 epitope records. (ii) Secondly, we converted the H1 numbering into that for all the other HA subtypes. This was done by inputting the H1 reference sequence into the HA Subtype Numbering Conversion tool in IRD, and then selecting all subtypes in the Conversion Sequence Numbering Scheme list. This tool BLASTs the input sequence against all HA reference sequences, returns the closest reference sequence (22) and then converts the input numbering into that for other subtypes. The HA Subtype Numbering Conversion Result page displays a coordinate mapping table as well as an alignment file for download (Figure 4A). (iii) Thirdly, we used the coordinate mapping table to map all H1 epitopes from step i to the reference strain of other subtypes. (iv) Lastly, for each epitope, we calculated the percent identity across the reference strains representing all subtypes. This analysis found that H1 B-cell epitopes in the HA stem are highly conserved across all subtypes; epitopes in the HA head are more variable in general, however, several epitopes in the head region are also conserved across all subtypes (Figure 4B). These conserved epitopes are candidate targets for eliciting a cross-reactive immune response.

## NEW DATA TYPES

### Linkouts to disease event data

To study virus evolution in the context of virus outbreaks, it is critical to have both epidemiological and genetic data. Since its inception, IRD has served as the repository for avian influenza virus surveillance data collected by the Centers of Excellence for Influenza Research and Surveillance (CEIRS) program. Concurrently, the Food and Agriculture Organization (FAO) of the United Nations receives data on worldwide animal disease events and provides access to such data through the Global Animal Disease Information System - EMPRES-i (23). In collaboration with EMPRES-i developers, IRD has now established links between strain and sequence records in IRD and disease event information in EMPRES-i. Such integration of surveillance and sequence data facilitates research on the evolution and molecular epidemiology of influenza viruses.

### Host factor data

A variety of different cellular proteins are utilized by viruses to facilitate viral replication. Conversely, other host factors function to sense the presence of viruses and prevent their further replication. There has been a growing interest in applying systems biology approaches to explore host-virus interactions to better understand the host responses to virus infections. Initially developed as a resource focused on virus data, IRD has expanded its scope to capture host factor data produced by the NIAID Systems Biology for Infectious Diseases Research program (2). As of July 2016, IRD provides access to 57 structured data sets regarding host responses to virus infection, among which 35 are related to influenza virus infection experiments. Currently supported experiment types include transcriptomic, proteomic and lipidomic experiments. The uniqueness of the IRD host factor component lies in: (i) providing standardized experiment and sample metadata, (ii) displaying transcriptional response patterns computed from an in-house statistical pipeline, (iii) enabling the comparison of host responses detected under different experimental and infection conditions, and (iv) supporting gene set enrichment analysis.

**Figure 5.**
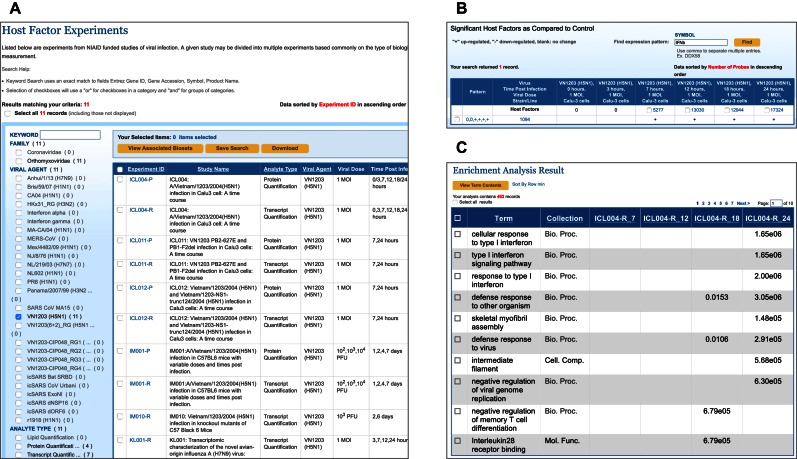
Host factor component in IRD. (**A**) A portion of the Host Factor Experiments page, showing a list of experiments using A/Vietnam/1203/2004 human isolate (VN1203 (H5N1)) as the viral agent. (**B**) The Host Factor Bioset Patterns table showing the expression pattern of IFNb in this experiment. (**C**) A portion of the Enrichment Analysis Result page displaying the terms, the collections (GO categories in this case) and the *P*-values calculated by the CLASSIFI algorithm using a hypergeometric distribution function.

To illustrate the functionality of the host factor component, we explored the host response patterns to H5N1 virus infection followed by gene enrichment analysis using the following workflow: (i) To begin, we searched for host factor experiments using VN1203 (H5N1) as the viral agent. As of July 2016, this query returned 11 experiments, including seven transcriptomic and four proteomic experiments (Figure 5A). (ii) From the returned experiment list, we selected experiment ‘ICL004-R’ to load the Experiment Details page. This page displays experiment information, an experiment sample summary, host factor bioset information, a host factor bioset summary, host factor bioset patterns and host factor results. Each bioset contains a list of host factors generated from a statistical comparison between virus infected and mock-infected samples. (iii) The Host Factor Bioset Patterns section of the Experiment Details page shows statistically-significant host factors grouped by expression patterns. For this use case, we searched for the expression pattern of interferon beta gene in this experiment by entering ‘IFNb’ into the Symbol search box and selecting ‘Find’. The expression pattern for IFNb was found to be ‘0,0,+,+,+,+’ (Figure 5B), indicating that transcription of IFNb was significantly upregulated during the latter 4 timepoints of the experiment. (iv) Next, we selected the hyperlinked host factor number to retrieve all host factors exhibiting the same expression pattern, together with the associated fold change and statistical support values. This host factor list can be saved to a working set in the workbench, downloaded to local computers, or transferred to pathway analysis tools. (v) In order to perform pathway enrichment analysis, we selected all host factors by ticking the ‘Select all’ box above the table, mousing over the ‘Run Analysis’ dropdown menu and selecting ‘Enrichment Analysis’. On the Enrichment Algorithm and Gene-Annotation Collection page, we selected Enrichment Algorithm—CLASSIFI, Gene-Annotation Collection—Gene Ontology (GO) and Gene-Annotation Background—From Experiment. The Enrichment Analysis Result page then gave the GO terms and associated *P*-values calculated by the CLASSIFI algorithm using a hypergeometric distribution function (Figure 5C) (24). One of the most significant biological processes enriched in this gene set is ‘cellular response to type I interferon’ as might be expected from an expression pattern that includes interferon beta. This workflow demonstrates the power of the host factor component and how it can be applied to better understand the host response to viral infection.

### Antiviral drug data

As IRD continues to grow and expand, a new focus area is to develop a comprehensive support infrastructure for antiviral drug data management and analysis. Most recently, the IRD development team has curated antiviral drug data related to influenza virus as well as other viruses supported in the Virus Pathogen Resource (www.viprbrc.org) (25), a sister resource of IRD. Current antiviral drug data includes descriptive drug information, 3D structures for drug/protein target complexes, specific drug interaction sites and antiviral resistance mutations.

## USER SUPPORT

To help the community utilize the wide range of functionalities provided in the resource, IRD provides on-site training workshops, demonstration workshops in conjunction with major scientific meetings, as well as online tutorials and training materials. In the past five years, IRD has sponsored on-site training workshops at 22 institutes including the NIH, US CDC, USDA, Harvard University, Massachusetts Institute of Technology, Chinese CDC, Chinese Academy of Sciences and Duke-NUS Graduate Medical School of Singapore, reaching approximately 600 users with hands-on training. Additionally, a Contact Us form is provided on the website to assist users with specific questions. Finally, all IRD protocols are described in SOPs on the website for users interested in the underlying computational details.

## FUTURE DEVELOPMENTS

Building upon the curated anti-viral drug data, IRD is in the process of expanding the antiviral drug component by: (i) curating drug interaction sites as Sequence Features and subsequently predicting whether a virus strain is likely to be drug resistant using our existing PVT computational pipeline, (ii) integrating host factor data with drug target data to facilitate the discovery of potential host factor drug targets, (iii) representing drug target data using the OpenBEL language and (iv) developing an anti-viral drug resistance risk assessment tool.

In the initial implementation, IRD integrated several Java-applets for various analysis and visualization tasks. With support for Java being phased out of some web browsers, these applets are being replaced by JavaScript alternatives. Recently IRD has implemented the JSMOL protein structure viewer as an alternative to JMOL. Replacements for the JalView sequence alignment viewer and Archaeopteryx tree viewer are currently under development.

As new variant proteins are identified by the influenza research community and reported in the scientific literature (26), the IRD team evaluates the strength of evidence for the presence and importance of these novel proteins, determines the sequence signals that can be used to predict their expression, and adds the specific prediction algorithm to our variant protein prediction infrastructure, making these sequence annotations uniquely available in IRD for user query and downstream analysis.

By continuing to expand data contents and analysis functionalities, IRD continues to provide a powerful bioinformatics resource for influenza virus data mining and hypothesis generation, thus expediting the research and development of diagnostics, vaccines and therapeutics against influenza virus.
